# Comparative Transcriptome Analysis between Resistant and Susceptible Pakchoi Cultivars in Response to Downy Mildew

**DOI:** 10.3390/ijms242115710

**Published:** 2023-10-28

**Authors:** Yaosong Chen, Liming Miao, Xiaofeng Li, Yiwen Liu, Dandan Xi, Dingyu Zhang, Lu Gao, Yuying Zhu, Shaojun Dai, Hongfang Zhu

**Affiliations:** 1 Shanghai Key Laboratory of Protected Horticultural Technology, Horticulture Research Institute, Shanghai Academy of Agricultural Sciences, Shanghai 201403, China; 17837194050@163.com (Y.C.);; 2 Development Center of Plant Germplasm Resources, College of Life Sciences, Shanghai Normal University, Shanghai 201418, China; 3 Institute of Agricultural Science and Technology Information, Shanghai Academy of Agricultural Sciences, Shanghai 201403, China

**Keywords:** pakchoi, downy mildew, transcriptome, differentially expressed genes, salicylic acid

## Abstract

Downy mildew caused by the obligate parasite *Hyaloperonospora brassicae* is a devastating disease for *Brassica* species. Infection of *Hyaloperonospora brassicae* often leads to yellow spots on leaves, which significantly impacts quality and yield of pakchoi. In the present study, we conducted a comparative transcriptome between the resistant and susceptible pakchoi cultivars in response to *Hyaloperonospora brassicae* infection. A total of 1073 disease-resistance-related differentially expressed genes were identified using a Venn diagram. The Gene Ontology and Kyoto Encyclopedia of Genes and Genomes pathway analyses revealed that these genes were mainly involved in plant−pathogen interaction, plant hormone signal transduction, and other photosynthesis-related metabolic processes. Analysis of the phytohormone content revealed that salicylic acid increased significantly in the resistant material after inoculation with *Hyaloperonospora brassicae*, whereas the contents of jasmonic acid, abscisic acid, and 1-aminocyclopropane-1-carboxylic acid decreased. Exogenous salicylic acid treatment also significantly upregulated *Hyaloperonospora brassicae*-induced genes, which further confirmed a crucial role of salicylic acid during pakchoi defense against *Hyaloperonospora brassicae*. Based on these findings, we suggest that the salicylic-acid-mediated signal transduction contributes to the resistance of pakchoi to downy mildew, and *PAL1, ICS1, NPR1, PR1, PR5, WRKY70, WRKY33, CML43, CNGC9,* and *CDPK15* were involved in this responsive process. Our findings evidently contribute to revealing the molecular mechanism of pakchoi defense against *Hyaloperonospora brassicae*.

## 1. Introduction

Pakchoi (*Brassica campestris ssp. chinensis Makino*), which originated in China, has become a popular leafy vegetable globally [1]. Downy mildew (DM) is caused by obligate parasitic oomycete *Hyaloperonospora brassicae* (*H. brassicae*). *H. brassicae* prefers low-temperature and high-humidity environments, which causes disease outbreaks, especially from late autumn to early spring each year in pakchoi [2]. DM often causes leaf wilting and even death of the entire pakchoi, and this restricts leafy vegetable cultivation and development in China [3]. Currently, the main approach to control DM is fungicide application, while it is usually poorly effective and even induces an emergence of fungicide-resistant mutants [4]. Therefore, identifying disease-resistant genes and cultivating disease-resistant cultivars are the fundamental ways to prevent DM in pakchoi.

To develop green and efficient control methods, it is indispensable to thoroughly understand the mechanism of disease resistance in plants. Plant innate immune systems can be classified into pathogen-associated molecular pattern-triggered immunity (PTI) and effector-triggered immunity (ETI) [5]. PTI is triggered by plant pattern-recognition receptors (PRRs) recognizing pathogen-associated molecular patterns (PAMPs). ETI is triggered by plant-disease-resistance proteins (R proteins) that recognize effectors produced by pathogens, which leads to the production of specific defense responses. ETI is an accelerated and amplified PTI effect [6], and its occurrence leads to plant disease resistance. Long-term natural selection would produce novel pathogenic bacteria which can escape from ETI while also stimulating new resistance genes to restart ETI [7]. Following plant infection with pathogens, multiple defense genes encoding pathogenesis-related proteins (PRs) are activated in the infected and uninfected areas, leading to systemic acquired resistance (SAR) [8]. Studies have shown that salicylic acid (SA) is an important signal activating PTI and ETI [9]. In addition to SA, jasmonic acid (JA), abscisic acid (ABA), and ethylene (ET) were also demonstrated to take part in regulating plant immunity [9,10].

The signal-transduction pathway mediated by SA is a complex network that involves multiple genes’ regulation. Following pathogen invasion, the upstream regulatory EDS1 and PAD4 lead to the accumulation of SA. Both EDS1 and PAD4 are simultaneously subjected to positive feedback regulation by SA [5]. The downstream signaling of SA is primarily regulated by NPR1. SA induces the formation of monomers of NPR1 from oligomers; SA-bound NPR1 enter into the nucleus to control the production of downstream resistance proteins, such as PRs induced by pathogens in infection [6]. Therefore, alteration of SA content and the expression of *PRs* involve the mechanism of plant disease resistance [7]. In this process, transcription factors, such as TGAs and WRKYs, interact with NPR1 to regulate the expression of downstream genes in the nucleus [8]. Previous research showed that the expression of major genes in the SA signaling pathways were induced after DM invasion in Chinese cabbage, which implied that SA plays a crucial role in DM resistance [9]. It is generally accepted that SA is involved in plant resistance against biotrophic and hemibiotrophic pathogens, while, the co-action of JA and ET signals contributes to plant resistance against necrotrophic pathogens [10]. Jing Li et al. proposed that cross-talk among different plant hormone-signaling pathways is a central feature of the plant defense-signaling network, with WRKY70 playing a crucial role in balancing between SA-dependent and JA-dependent defense pathways [11].

Previous studies on DM have focused on the molecular mechanisms of plant–pathogen interactions. Asai et al. found that effectors of Arabidopsis DM escape recognition through expression of polymorphism and subcellular localization [12]. The DM effector proteins ATR1 and ATR13 promote susceptibility to the disease in Arabidopsis [13]. Additionally, Mahmut et al. provided a detailed description of evolution and classification of DM pathogens [14]. Currently, cucumber and grapevine are the crops that have received more research attention in terms of combating DM stress. The research has mainly concentrated on the screening and validation of resistance genes. Shuangshuang Yan et al. identified the cucumber resistance gene *CsIVP* against DM. CsIVP negatively regulates the synthesis of SA, and together with CsNIMIN1 inhibits the expression of *PR1*, thereby reducing the cucumber’s resistance to DM [15]. VvNAC72 in grapevine suppresses VvGLYI-4, a negative regulator of methylglyoxal (MG) detoxification, and regulates the dynamic balance of MG-related ROS through the SA-mediated defense pathway, ultimately enhancing resistance against DM [16]. Tingting Gao et al.’s research on resistant cabbage revealed that the resistance mechanism against DM follows a gene-for-gene model, where disease-resistance genes mainly activate the SA signaling pathway to prevent pathogen colonization. However, the involvement of basal defense mediated by JA and ET cannot be ruled out in DM resistance [9]. Bin Zhang et al. successfully localized the DM resistance gene *BrWAK1* in cabbage. Both SA and DM inoculation induce the expression of *BrWAK1*; overexpression of *BrWAK1* significantly enhances resistance to DM in susceptible materials, while silencing *BrWAK1* in resistant materials increases susceptibility [17]. Although some research on DM has been reported, the mechanism of disease resistance is not yet fully understood in pakchoi.

In the present study, we examined the transcriptional response of pakchoi to *H. brassicae* infection using RNA-seq, identified differentially expressed genes (DEGs) between resistant and susceptible materials induced by *H. brassicae* infection, and analyzed the change of hormone contents and the associated resistance pathways in response to *H. brassicae*. We have discovered that SA content and the expression of key genes involved in the SA-signal-transduction pathway were induced by *H. brassicae* in pakchoi. Importantly, our findings reveal a similarity in the response of Chinese cabbage [9] and grapevine [18] to DM. These identified genes are hypothesized to confer resistance against DM in this context. Collectively, our findings provide important basic messages related to the DM resistance of pakchoi, which will contribute to breeding disease-resistant varieties in the future.

## 2. Results

### 2.1. Phenotypic Identification of the Resistant Inbred Line (R) and the Susceptible Line (S)

**Figure 1:**
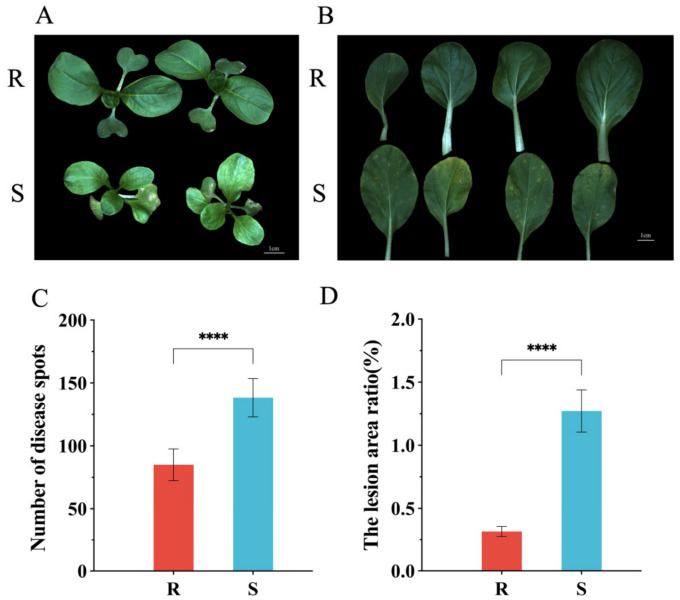
Differential phenotypes of R and S seedlings upon *H. brassicae* treatment. (**A**) Growth vigor of R and S after being invaded by *H. brassicae*. (**B**) Leaf surface of R and S after being invaded by *H. brassicae*. (**C**) The number of disease spots on leaves of R and S after 14 days. (**D**) The ratio of disease-spot area on leaves of R and S after 14 days. Error bars represent ± SD (*n* = 8 biological replicates); “****” represents *p* < 0.0001.

Pakchoi leaves showed extensive yellow spots on the leaf surface and reduced growth vigor after being invaded by *H. brassicae* (Figure 1A). We inoculated *H. brassicae* on R and S, respectively, at the seedling stage and observed the number of disease spots on leaves of R and S after 14 days (Figure 1B). Four randomly selected leaves from each group were used to calculate the number of disease spots and the ratio of disease-spot area to leaf area. The number of disease spots and the ratio of disease-spot area in R were significantly lower than those in S (Figure 1C,D). Therefore, we identified R and S as the resistant and susceptible materials, respectively.

### 2.2. Quality Evaluation and Analysis of RNA-Seq Data

**Figure 2:**
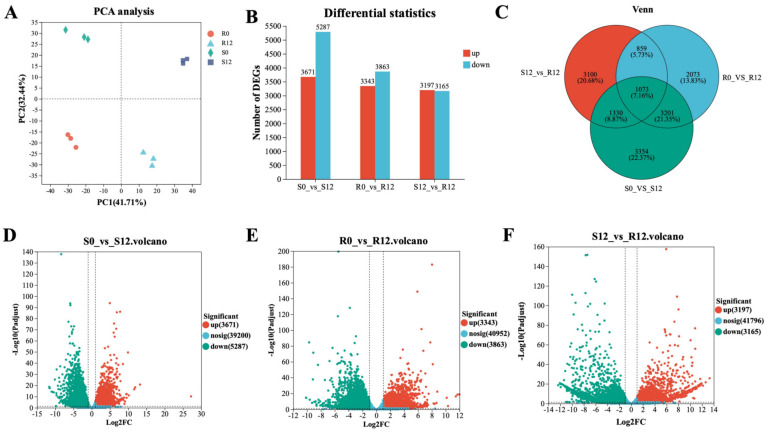
An overview of the RNA-seq data for disease-resistant and susceptible materials during the 0 and 12 hpi phases. (**A**) Principal Component Analysis (PCA) of RNA-seq data. (**B**) The number of Differentially Expressed Genes (DEGs) at different stages in disease-resistant and susceptible materials. Red bars indicate upregulated genes and blue bars indicate downregulated genes. (**C**) Venn diagram of DEGs in three comparisons. (**D**–**F**) The *x*-axis represents the fold change in gene expression. The *y*-axis represents the statistical test value (*p*-value) for the difference in gene expression. Both the *x*-axis and *y*-axis values have been logarithmically transformed. Each point in the graph represents a specific gene, with red points indicating significantly upregulated genes, green points indicating significantly downregulated genes, and blue points representing genes with non-significant differences in expression. (**D**) Volcano analysis indicating the DEGs between S0 and S12. (**E**) Volcano analysis indicating the DEGs between R0 and R12. (**F**) Volcano analysis indicating the DEGs between S12 and R12.

RNA-seq analysis was conducted on both disease-resistant and susceptible materials at 0 h and 12 h after inoculation with *H. brassicae*, yielding a total of 91.07 Gb of clean data with a Q20 base percentage >98.61% and GC content in the 47.58–48.56% range. The clean reads were blasted to the reference genome of pakchoi [19], with alignment rates ranging from 88.41% to 90.64% (Table S1). Based on the expression matrix, Principal Component Analysis was performed on the 12 samples, results showed good within-group repeatability and between-group variability (Figure 2A). PCA of 12 sample groups also revealed significant differentiation between resistant and susceptible materials. The resistant and susceptible materials exhibited heterogeneity at different stages after inoculation with *H. brassicae*. Overall, the results demonstrate that high-quality RNA-seq data were obtained for the present study.

To identify genes responsive to *H. brassicae* infection in Pakchoi, we compared R and S at 12 h post-infection (hpi) with *H. brassicae* to their respective 0 hpi (R0_VS_R12, S0_VS_S12). Additionally, we compared the differences between R and S after being inoculated with *H. brassicae* (S12_VS_R12) to screen for disease-resistant genes (Figure 2B). Compared with the S-0 hpi, 8958 DEGs were identified at 12 hpi, with 3671 upregulated and 5287 downregulated genes detected in the susceptible-material leaves after being infected with *H. brassicae* (Figure 2D). Compared with R-0 hpi, 7206 DEGs were detected, including 3343 upregulated and 3863 downregulated genes at 12 hpi in the resistant-material leaves after being infected with *H. brassicae* (Figure 2E). Compared with S-12 hpi, 6362 DEGs including 3197 upregulated genes and 3165 downregulated genes were identified at the R-12 hpi (Figure 2F). The Venn diagram showed that 1073 genes were the common DEGs among R0_VS_R12, S0_VS_S12, and S12_VS_R12, which will be the focus of our attention in follow-up research (Figure 2C).

### 2.3. Gene Ontology Analysis of the 1073 DEGs

**Figure 3:**
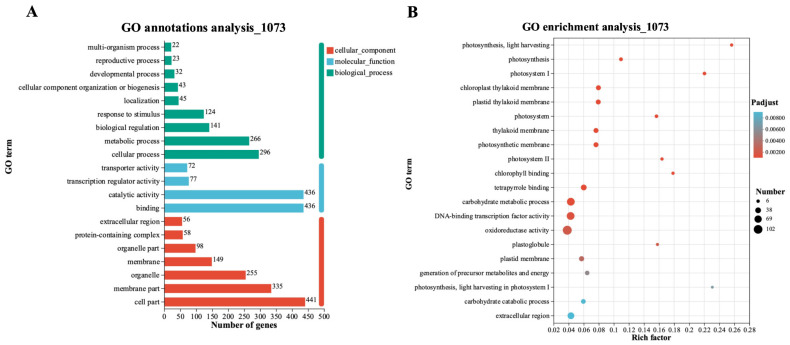
GO-function classification analysis of DEGs. (**A**) Numbers of DEGs in three ontologies, including molecular function, cellular component, and biological process. (**B**) GO enrichment analysis of DEG, and the list of top-20 GO terms calculated with the *p*-adjust.

To identify the functions of DEGs induced by *H. brassicae*, Gene Ontology (GO) classification was performed with the 1073 DEGs. GO analysis showed that *H. brassicae* induced the expression of genes related to different functions, especially “metabolic process” and “cellular process” in the biological processes (BP) ontology; “catalytic activity” and “binding” in the molecular functions (MF) ontology; and “membrane part” and “cell part” in the cell components (CC) ontology (Figure 3A). In the BP category, the highest number of DEGs was enriched in the “cellular process”; the highest number of DEGs in the MF category was enriched in the “cell part”. In the CC category, the highest number of DEGs was observed in “binding”. Among the top 20 abundant GO terms, the most significant were for photosynthesis, the light-harvesting pathways, and most of them were related to photosynthesis (Figure 3B). A total of 10 terms were enriched in cellular component ontology, 6 terms from biological process ontology were enriched, and only 4 terms were enriched in molecular function ontology. Thus, the GO enrichment analysis indicated that *H. brassicae* infection had a greater influence on the cellular component compared with the biological process and the molecular function.

### 2.4. Kyoto Encyclopedia of Genes and Genomes Pathway-Enrichment Analysis of the 1073 DEGs

**Figure 4:**
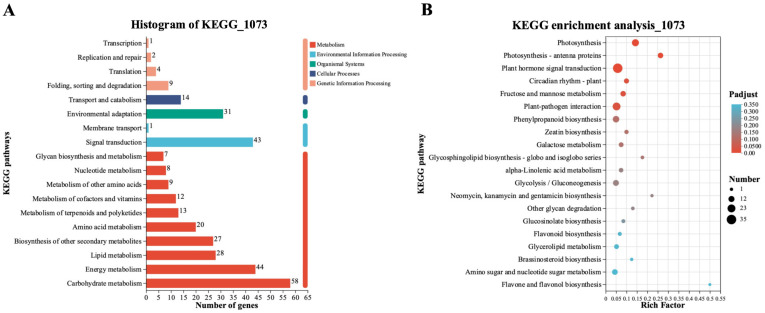
KEGG pathway-enrichment analysis of 1073 DEGs. (**A**) KEGG histogram of 1073 DEGs; the ordinate is the name of the KEGG metabolic pathway; and the abscissa is the number of genes or transcripts annotated to that pathway. (**B**) The list of the top-20 KEGG pathways calculated using the Q-value. The size of the dot indicates the quantity; the redder the color, the smaller the *p*-adjust. The *p*-adjust is the multiple-hypothesis test-corrected *p*-value.

The Kyoto Encyclopedia of Genes and Genomes (KEGG) pathway-enrichment analysis was further used to identify the metabolic pathways which were involved in response to *H. brassicae* of pakchoi. As shown in Figure 4A, 1073 DEGs were classified into 18 categories. A total of 10 terms were enriched for metabolism, 4 terms from genetic-information processing were enriched, and 2 terms were enriched in environmental-information processing. Only one term was enriched in cellular processes and organic systems, respectively. Analysis of the top-20 enriched pathways showed that photosynthesis, photosynthesis-antenna proteins, plant hormone signal transduction, circadian rhythm of plant, fructose and mannose metabolism, and plant–pathogen interaction, etc., were significantly induced by DM in pakchoi (Figure 4B). It is worth noting that “plant hormone signal transduction” was enriched with the highest number of genes. In addition to plant hormone signal transduction, 23 DEGs were enriched in the plant–pathogen interaction pathway. These results imply that *H. brassicae* infection activates the phytohormone-mediated pathogen-defense process in pakchoi. We further focused on analyzing the DEGs which are involved in plant hormone signal transduction and plant–pathogen interaction pathways in the following exploration.

### 2.5. Analysis of SA Biosynthesis and Signal Transduction after Inoculation of H. brassicae in Pakchoi

According to the results of enrichment analysis based on the KEGG database, many DEGs were involved in the “plant hormone signal transduction” pathway, including those mediated by SA, JA, ET, and ABA in response to *H. brassicae* infection, so we measured phytohormone levels of the resistant material at 0, 6, and 12 hpi (Figure S1). Compared with the control group, SA content was significantly upregulated after inoculation of *H. brassicae,* and its level reached to peak at 6 hpi; SAG content also showed a similar accumulation trend, while JA and ABA contents decreased after the inoculation of *H. brassicae*. ET precursor 1-aminocyclopropane-1-carboxylic acid (ACC) content was higher at 12 hpi than at 0 hpi, but this result was not significant. These results indicate that *H. brassicae* induces SA biosynthesis and signal transduction, thus initiating an immunoreaction of pakchoi.

**Figure 5:**
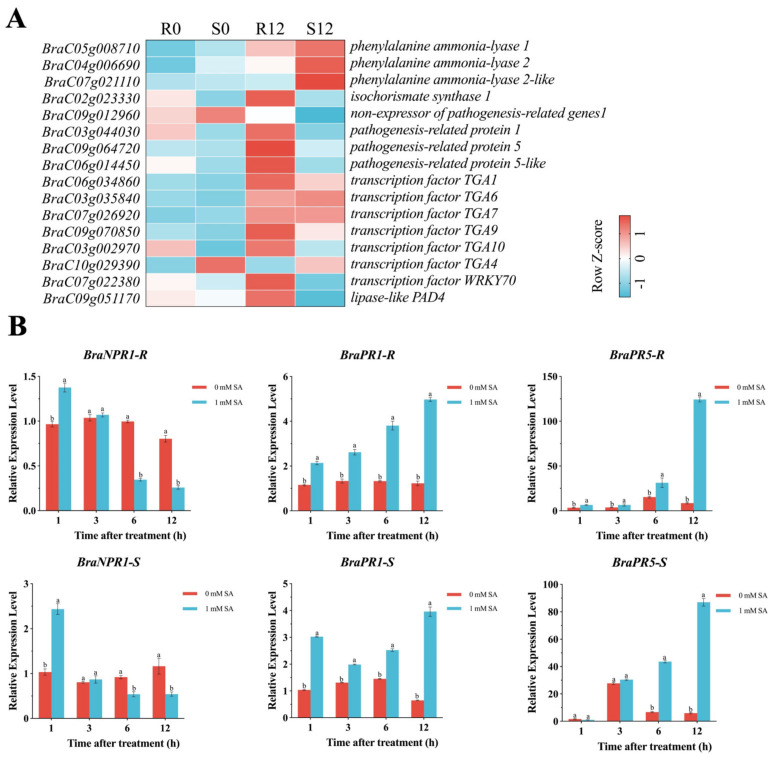
SA biosynthesis and expression changes of response genes to *H. brassicae*. Exogenous SA treatment confirmed the downstream regulatory genes of the SA signaling pathway in pakchoi. (**A**) Heatmap of DEGs related to SA biosynthesis and signaling in R0, S0, R12, and S12. (**B**) After treating pakchoi leaves with exogenous 1 mM SA, the expression trends of *BraNPR1* (*BraC09g012960*)*, BraPR1* (*BraC03g044030*), and *BraPR5* (*BraC09g064720*) were analyzed. Error bars represent the average value ± standard deviation from three biological replicates. The horizontal axis represents the time of exogenous SA treatment. Letters indicate significant differences (*p* < 0.05).

We further focused on the transcriptional levels of key genes in the SAR-signal-transduction pathway regulated by SA (Figure 5A). Plants synthesize SA through two pathways: the isochorismate synthase pathway and the phenylalanine ammonia-lyase pathway. In this study, the expression of *PAL1 (BraC05g008710)* and *ICS1 (BraC02g023330)* in R12 was 6.3- and 1.9-fold higher than that in R0, respectively. The expression of *ICS1 (BraC02g023330)* was upregulated after stimulation by *H. brassicae*, with the resistant material showing 6.0-fold higher expression compared to the susceptible material at 12 hpi. As the receptor of SA, *NPR1 (BraC09g012960)* in resistant material was higher than that in the susceptible material at 12 hpi. Similarly, the expression of three *PRs* (*BraC03g044030*, *BraC09g064720*, *BraC06g014450*) were significantly upregulated in the resistant material at 12 hpi. *TGAs*, encoding TFs interacting with NPR1 to regulate target genes, were also upregulated with the inoculation of *H. brassicae*. *PAD4*, which was regulated by the SA signal, was more accumulated in the resistant material than in the susceptible material. *WRKY70,* which functions as an activator of SA-dependent defense genes showed an 11.2-fold upregulation at 12 hpi in the resistant material. These findings indicate that the SA signaling pathway is involved in the regulation of the defense of *H. brassicae* in pakchoi.

Treating the leaves of resistant and susceptible materials with 1 mmol·L^−1^ SA or 0 mM SA (control) and analyzing the expression pattern of *NPR1*, *PR1*, and *PR5* showed that, compared to the control samples, *NPR1* was significantly upregulated after 1 h treatment of the SA in resistant and susceptible materials. *PR1* was significantly upregulated after SA treatment, and the greatest difference between the control and treatment was at 12 h. Expressions of *PR1* and *PR5* showed similar changes in the two materials. Exogenous SA treatment induced a significant upregulation of *PR5*, with the highest difference being observed at 12 h in the resistant materials. While in the susceptible materials, *PR5* upregulated was significantly upregulated at 6 h and 12 h (Figure 5B). The upregulation of *PRs* induced by exogenous SA confirmed that *PRs* are downstream genes in the SA-mediated disease-resistance pathway in pakchoi. Collectively, *H. brassicae* induced an increase in SA level and its downstream genes, which highlights that SA plays a crucial role in contributing to pakchoi defense against DM.

### 2.6. Analysis of the Differentially Expressed Genes Involved in Disease Resistance in the Plant–Pathogen Interaction Pathway

**Figure 6:**
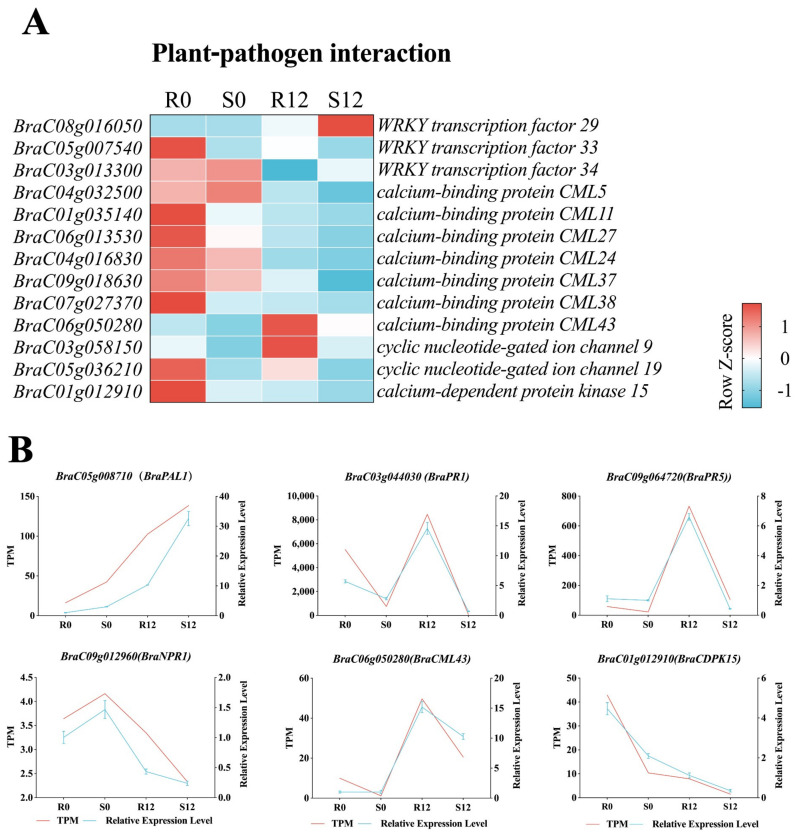
DEGs that may be involved in disease resistance in the plant–pathogen interaction pathway. Quantitative real-time PCR of genes related to the resistance pathway against *Hyaloperonospora brassicae* infection in pakchoi. (**A**) Heatmap of DEGs related to plant–pathogen interaction in R0, S0, R12, and S12. (**B**) Comparison of gene-expression patterns between RNA-seq and qRT-PCR.

According to the enrichment results, we further chose the enrichment pathway of plant–pathogen interaction for further investigation. As shown in Figure 6A, compared with 0 hpi, the expression of *CNGC9* (*BraC03g058150*) was induced by *H. brassicae* and significantly increased in resistant and susceptible materials at 12 hpi. Simultaneously, the expression of *CNGC9* in resistant materials was significantly higher than that in susceptible materials at 12 hpi. The expression of *CML43* (*BraC06g050280*) showed a similar expression pattern. In addition, a variety of calmodulin-like proteins, such as *CML5*, *CML11*, *CML24*, *CML27*, *CML3*7, and *CML38* were enriched in the plant–pathogen interaction term. The expression of *CDPK15* (*BraC01g012910*), *WRKY33* (*BraC05g007540*), and *CNGC19* (*BraC05g036210*) were significantly downregulated after being infected by *H. brassicae*, but the expression in resistant materials was significantly higher than that in susceptible materials at 12 hpi. We randomly selected 6 DEGs [*BraC05g008710(BraPAL1)*, *BraC03g044030(BraPR1)*, *BraC09g064720(BraPR5)*, *BraC09g012960(BraNPR1)*, *BraC06g050280(BraCML43)*, and *BraC01g012910(BraCDPK15)*] from the transcriptome results for qRT-PCR analysis to verify the RNA-seq data (Figure 6B). The qRT-PCR results showed that the expression patterns of the 6 DEGs were consistent with the RNA-seq results, demonstrating that the RNA-seq data is correct.

## 3. Discussion

DM is a common and serious oomycete disease in Brassicaceae crops, and seriously affects the development of leafy vegetable production. Therefore, establishing green and efficient control measures for this disease is indispensable while research related to *H. brassicae* in pakchoi is limited. Identifying resistant genes and pathways could facilitate the breeding of resistant varieties. In this study, we obtained 1073 DEGs correlated with the DM-resistant process using Venn analysis (Figure 2C). These genes were significantly induced by *H. brassicae* and expressed differently in resistant and susceptible materials. GO and KEGG enrichment analysis showed that these 1073 DEGs were highly enriched in photosynthesis, plant–pathogen interaction, plant hormone-signal-transduction pathways, etc. (Figure 4B). Previous studies highlighted that SA, JA, and ET are the main phytohormones participating in disease resistance in plants [20]. In this study, the SA content was significantly upregulated at 6 hpi (consistent with the upregulation of *PAD4* which regulates SA accumulation), and the level of other phytohormones either did not change considerably or declined following infection with *H. brassicae* (Figure S1). In addition to SA biosynthesis, the SA-response genes were also induced with *H. brassicae* in pakchoi (Figure 5B). The expression of *NPR1*, *PR1*, and *PR5* showed an upward trend upon exogenous SA treatment, indicating that *H. brassicae* activates the defense system of pakchoi and the SA-mediated disease-resistance pathway plays a major role in this defense process. Previous studies reported that the defense response of grapes, canola [21], Arabidopsis [22], cucumber [23], spinach [24], and lychee [25] against DM is predominantly mediated by SA [26]. These results are consistent with our results that SA plays a crucial role in regulating plants’ resistance against DM.

PRs were first discovered in the tobacco mosaic-virus-infected leaves [27]. *PR1* is induced during SA-dependent defense responses, and it was used as a defense-marker gene [28]. In the present study, the expression of *PR1* in resistant material was considerably upregulated at 12 hpi (Figure 5A). *PR1* enhances the resistance of plants to bacteria [29], fungi [30], oomycetes [31], and other diseases. Zaynab et al. found that PR1 plays a key role in potato resistance against *Phytophthora infestans* [32]. Gamir et al. found that PR1 (P14c and PR1a) from tomato and tobacco inhibits the growth of *Plasmodiophora brassicae* (oomycete) in Brassicaceae plants [33]. Although some studies have demonstrated that *PR1* has anti-oomycete disease function, no reports have investigated its relationship with DM. Based on the expression patterns of *PR1* in pakchoi, we hypothesize that *PR1* responds favorably to DM and acts as a candidate-resistant gene aiding against DM in pakchoi.

The plant–pathogen interaction mechanism is crucial for plant response to pathogen stress. In this study, seven *CMLs*, two *CNGCs*, one *CDPK*, and three *WRKYs* were identified, which were involved in *H. brassicae* stress response and differentially expressed in resistant and susceptible materials (Figure 6A). CNGCs were a type of non-specific cation channel, which was an important way for plants to resist against the invasion of pathogens by regulating Ca^2+^ influx [34]. Compared with the susceptible materials, we found the expression of *CNGC9* and *CNGC19* were significantly higher than that in the resistant materials at 12 hpi. Previous studies showed that *AtCNGC19* is crucial in the perception of herbivory by regulating the Ca^2+^ fluxes [35]; *CNGC11* and *CNGC12* positively regulate Arabidopsis resistance to *H. brassicae* and bacteria [36,37,38]; CDPKs have emerged as important Ca^2+^ sensor proteins in transducing differential Ca^2+^ signatures; and, when plants are under stress, calcium ions inside cells form specific calcium-ion signals that bind to CMLs and activate CDPKs. CDPKs triggered by PAMPs or effectors activate complex downstream responses [39]. Previous research showed that overexpression of *CML43* in Arabidopsis accelerates a hypersensitive reaction after infection with *Pseudomonas syringae* [40], and *CML9* is involved in regulating plant immune response to pathogenic microorganisms [41]. After inoculation with *H. brassicae*, the expression of *CML43* significantly increased and the increase was greater in the resistant material. *CDPK15* may be involved in negative feedback regulation, as the expression level decreased suddenly in both materials. We speculate that *CDPK15* may play a role in calcium-signal recognition and transduction in the interaction between pakchoi and DM. *CNGC9* expression increased following inoculation with *H. brassicae*, and the expression in the resistant material was higher than that in the susceptible material, suggesting that *CNGC9* positively regulates the resistance of pakchoi to DM. In summary, members of the CDPKs, CMLs, and CNGCs participate in the generation of calcium signals and the regulation of gene expression during the immune response of pakchoi to DM.

WRKY is one of the largest transcription-factor families in higher plants. WRKYs also play a crucial role in plant immunity [42]. It has been shown that several WRKYs take part in the activation of plant defense reactions against various pathogens [43]. There have been some reports on the response of WRKY to *H. brassicae*. It has been demonstrated that overexpression of *VvWRKY1* in transgenic tobacco enhances resistance to Phytophthora, downy mildew, and powdery mildew [44]. In this study, both the SA signaling pathway and the plant–pathogen interaction pathway enriched the *WRKY* transcription factors in the DM-infected samples (Figure 6A). Among them, *WRKY33* showed downregulation at 12 hpi, implying a negative regulatory role of WRKY33 in the defense response of pakchoi against *H. brassicae*. Furthermore, WRKY33 has been demonstrated as a negative regulator of the SA-triggered response, enhancing the sensitivity of a plant to the biotrophic pathogen *Pseudomonas syringae* [45]. In addition to *WRKY33*, *WRKY70* is significantly upregulated in resistant materials. As reported previously, WRKY70 was identified as a node between SA-mediated and JA-mediated signals in plant-defense pathogens [46]. The expression of *AtWRKY70* is activated with SA and inhibited by JA. In our study, we observed an increase in SA content and a decrease in JA content in the resistant materials at 6 hpi (Figure S1), which is consistent with the changes in *WRKY70* expression. This indicates that although the JA content was downregulated in pakchoi after infection of *H. brassicae*, the involvement of the JA-mediated signaling pathway in defense cannot be excluded. Therefore, further investigation is warranted to explore the synergistic effects of SA and other phytohormones during the defense process.

## 4. Materials and Methods

### 4.1. Plant Materials and Sample Preparation

The resistant inbred line (R) and the susceptible line (S) used in the present study were bred through multiple generations of self-pollination by our research group. All materials were planted in the greenhouse at the Huacao campus of the Shanghai Academy of Agricultural Sciences, Shanghai, China, on 10 December 2021.

The diseased leaves were washed with distilled water, wrapped in wet filter paper, and placed in a culture dish. After dark treatment at 18 °C and 95% humidity for 24 h, spores that had grown out of the infected leaves were brushed into a suspension prepared with distilled water using a brush, ensuring a concentration of 4–5 sporangia per 10 × 10 field of view, and 0.1% Tween 20 was added as spore suspension.

The experimental materials were inoculated with *H. brassicae* using the spreading method on 24 December 2021. Uniformly growing and healthy experimental materials were selected, and 200 µL of spore suspension was evenly spread on the leaves. After inoculation, the materials were kept in the dark for 24 h at a temperature of 18–20 °C and a humidity of 95%. Samples of the resistant and susceptible materials were collected at 0 and 12 h post-infection, with three biological replicates for each time point. A total of 12 samples were used for subsequent transcriptome sequencing. They are, respectively R_0_1, R_0_2, R_0_3, S_0_1, S_0_2, S_0_3, R_12_1, R_12_2, R_12_3, S_12_1, S_12_2, and S_12_3. Symmetrical leaves from the whole plant were immediately collected and placed in liquid nitrogen and stored at −80 °C for subsequent analyses.

### 4.2. RNA Extraction, Library Construction, and Sequencing

Total RNA was extracted from the samples using TRIzol (Invitrogen, Waltham, MA, USA), and genomic DNA was removed using DNase I (TaKaRa). The quality of the RNA samples was determined using both 2100 Bioanalyzer (Agilent, Santa Clara, CA, USA) and ND-2000 (NanoDrop Technologies, Wilmington, DE, USA). The TruSeq mRNA sample preparation kit (Illumina, San Diego, CA, USA) was used to construct RNA libraries. The first strand of cDNA was synthesized, followed by second-strand synthesis to form a stable double-stranded structure. High-throughput sequencing was carried out using the Illumina HiSeq X Ten/NovaSeq 6000 sequencing platform. The result of the sequencing is paired-end and 150 bp read length. The datasets presented in this study can be found in online repositories. The names of the repository/repositories and accession number(s) can be found below: https://www.ncbi.nlm.nih.gov (accessed on 28 August 2023), PRJNA987043. Shanghai Majorbio Technology Co., Ltd. (Shanghai, China) conducted the entire sequencing procedure.

### 4.3. Transcriptome Data Analysis

We performed quality control on the raw sequencing data using SeqPrep 1.3.2-8 (https://github.com/jstjohn/SeqPrep, accessed on 28 August 2023) and Sickle 1.33+git20150314.f3d6ae3-2 (https://github.com/najoshi/sickle, accessed on 28 August 2023). Clean reads were aligned to the reference genome to obtain mapped reads using HiSat2 (http://ccb.jhu.edu/software/hisat2/index.shtml, accessed on 28 August 2023). The quality assessment of mapped reads was conducted using StringTie (https://ccb.jhu.edu/software/stringtie/index.shtml?t=example, accessed on 28 August 2023), which includes sequencing saturation, gene coverage, distribution of reads across different regions of the reference genome, and distribution of reads across different chromosomes.

To identify DEGs between two distinct samples, the expression level of each transcript was calculated using the transcripts per million reads (TPM) method. RSEM (http://deweylab.biostat.wisc.edu/rsem/, accessed on 28 August 2023) was used for the quantification of gene abundance. Differential expression analysis was performed using DESeq2 with a Q-value threshold of ≤0.05. Genes with |Log2FC| > 1 and Q-value ≤ 0.05 (DESeq2 or Edger) or Q-value ≤ 0.001 (DEGseq) were considered significantly differentially expressed genes.

Furthermore, functional enrichment analysis, including GO and KEGG, was performed to identify significantly enriched GO terms and metabolic pathways among the differentially expressed genes compared to the whole transcriptome background with Bonferroni-corrected *p*-value ≤0.05. GO functional enrichment and KEGG pathway analysis were conducted using Gotools 2.0.3 (https://github.com/tanghaibao/Goatools, accessed on 28 August 2023) and Kobas 3.0 (http://kobas.cbi.pku.edu.cn/home.do, accessed on 28 August 2023) tools, respectively.

### 4.4. Hormone-Content Detection

The quantification of six hormones, including salicylic acid (SA), SA-2-O-β-glucoside (SAG), jasmonic acid (JA), Jasmonoyl-L-Isoleucine, abscisic acid (ABA), and 1-aminocyclopropane-1-carboxylic acid (ACC), was conducted by Wuhan Metware Biotechnology Co., Ltd. (Wuhan, China) using Izumi et al.’s tandem mass spectrometry coupled to liquid chromatography (LC-MS/MS) platform. After grinding 50 mg of samples in liquid nitrogen, 10 μL of a 100 ng/mL internal standard mixture solution and 1 mL of extraction solvent containing methanol/water/formic acid (15:4:1, *v*/*v*/*v*) were added to the samples and mixed. The mixture was vortexed for 10 min, centrifuged at 12,000 rpm and 4 °C for 5 min, and the supernatant was collected and concentrated before being reconstituted with 80% methanol/water solution. After passing through a 0.22-μm filter membrane, the samples were placed in injection vials for LC-MS/MS analysis. The UPLC ExionLC™ AD and MS/MS QTRAP^®^ 6500+ system (AB Sciex Germany GmbH, Darmstadt, Germany) were used for data acquisition. The Metware Database (MWDB) was utilized for qualitative analysis of mass-spectrometry-detection data. Quantitative analysis was performed using the multiple reaction-monitoring mode of triple-quadrupole mass spectrometry.

### 4.5. Exogenous Salicylic Acid Treatment

To investigate the effects of exogenous SA treatment. After two weeks of planting, the resistant materials and susceptible materials were sparged with SA at thicknesses of 1 mmol·L^−1^. Distilled water was used as a control (0 mM SA). The treated samples were placed in a culture box with a temperature of 18 °C and a humidity of 60%. With three biological replicates for each time point, samples were obtained at 1, 3, 6, and 12 hpi. The samples were kept cold until analysis, frozen in liquid nitrogen, and stored at −80 °C.

### 4.6. qRT-PCR Evaluation of Candidate Genes

Six DEGs were arbitrarily selected from a variety of disease-resistant pathways and confirmed using a quantitative real-time polymerase chain reaction (qRT-PCR) to confirm the accuracy of the RNA-seq results. The β-actin gene of pakchoi was used as the reference gene, and qRT-PCR primers were designed using the https://primer3.ut.ee (accessed on 28 August 2023) website and synthesized by Qingke Biological Technology Co., Ltd. (Beijing, China) (Table S2). PrimeScript RT Master Mix kit (TaKaRa Bio Inc., Shiga, Japan) was used to reverse-transcribe RNA into cDNA. According to the manufacturer’s guidelines, qRT-PCR was carried out using the TransStart^®^ Top Green qPCR SuperMix kit (Quantiful GeneTech Co., Ltd., Beijing, China). The 2^−∆∆CT^ approach was used to determine the levels of expression of the target genes [47]. GraphPad Prism (9.4.1; GraphPad Software Inc., San Diego, CA, USA) was used to plot charts and graphs.

## 5. Conclusions

In the present study, we compared the transcriptome alterations between the resistant and susceptible pakchoi cultivars before and after *H. brassicae* infection. Our results revealed that the expression levels of SA biosynthesis and regulatory-related genes were upregulated by *H. brassicae*, with significantly higher expression levels observed in the resistant materials compared with the susceptible materials. Exogenous SA treatment confirmed the downstream regulatory genes of the SA signaling pathway in pakchoi. These findings also suggest that *PAL1, ICS1, NPR1, PR1, PR5, WRKY70, WRKY33, CML43, CNGC9,* and *CDPK15* act as the candidate genes responsible for the higher resistance to DM in resistant materials, which serve as the potential target genes contributing to breeding DM-resistant pakchoi.

## Data Availability

The datasets presented in this study can be found in online repositories. The names of the repository/repositories and accession number(s) can be found below: https://www.ncbi.nlm.nih.gov, PRJNA987043.

## Supplementary Materials

The following supporting information can be downloaded at: https://www.mdpi.com/article/10.3390/ijms242115710/s1.
