# Supplementary material for: Comparative Transcriptome Analysis between Resistant and Susceptible Pakchoi Cultivars in Response to Downy Mildew
Source: Int J Mol Sci. 2023 Oct 28;24(21):15710. doi: 10.3390/ijms242115710 (PMC10649052; doi:10.3390/ijms242115710)
Supplement: Supplementary file 1 [file ijms-24-15710-s001.zip › ijms-2608790-supplementary.pdf]

Table S1.

RNA-seq reads and mapping rate of different samples from non-heading Chinese cabbage leaves under *H. parasitica* inoculation.

Sample	Total reads	Q20(%)	GC content(%)	Multiple mapped	Uniquely mapped	Mapping rate(%)
R_0_1	51686204	98.61	48.37	2848241	44000481	90.64
R_0_2	49402294	98.74	47.73	2514839	41984563	90.08
R_0_3	51238984	98.71	48.31	3137953	42917407	89.88
S_0_1	57361484	98.77	48.56	2909863	49062344	90.6
S_0_2	59026454	98.74	48.27	3640286	48613146	88.53
S_0_3	51899286	98.7	48.5	2913196	43994657	90.38
R_12_1	54281584	98.73	47.81	2850513	46111803	90.2
R_12_2	63256426	98.68	47.68	3537529	53372804	89.97
R_12_3	46262670	98.63	47.98	2584492	38816735	89.49
S_12_1	48208244	98.72	47.58	2082757	40740438	88.83
S_12_2	46328742	98.73	47.65	1987840	39569639	89.7
S_12_3	48650266	98.75	47.72	2327205	40685472	88.41

Table S2.

The information of the primers used for qRT-PCR of 6 genes in this study.

Gene ID	Forward Primer （5’-3’）	Reverse Primer（5’-3’）
BraC03g044030	CCTACGCTCAAAACTACGCC	GCTTTGCCACATCCGATTCT
BraC05g008710	CATGTTCGCTCAGTTCTCCG	ACGTCTTGGTTGTGTTGCTC
BraC09g064720	CGGTTACAACGTCCAGATGG	ACAAGTCGCCGATGTACTGA
BraC09g012960	AAGTCATTGCCGCAACACAT	GTGGCCTTCAGTCAAAAGCA
BraC06g050280	GCGGATATCTCGGACCTCAA	GGAGATAAACCCGTCACCGT
BraC01g012910	TTGGACCGAGATGAGCACTT	GTCTTCCCTTGTTGTGGCTG
Actin	CTCAGTCCAAAAGAGGTATTCT	GTAGAATGTGTGATGCCAGATC

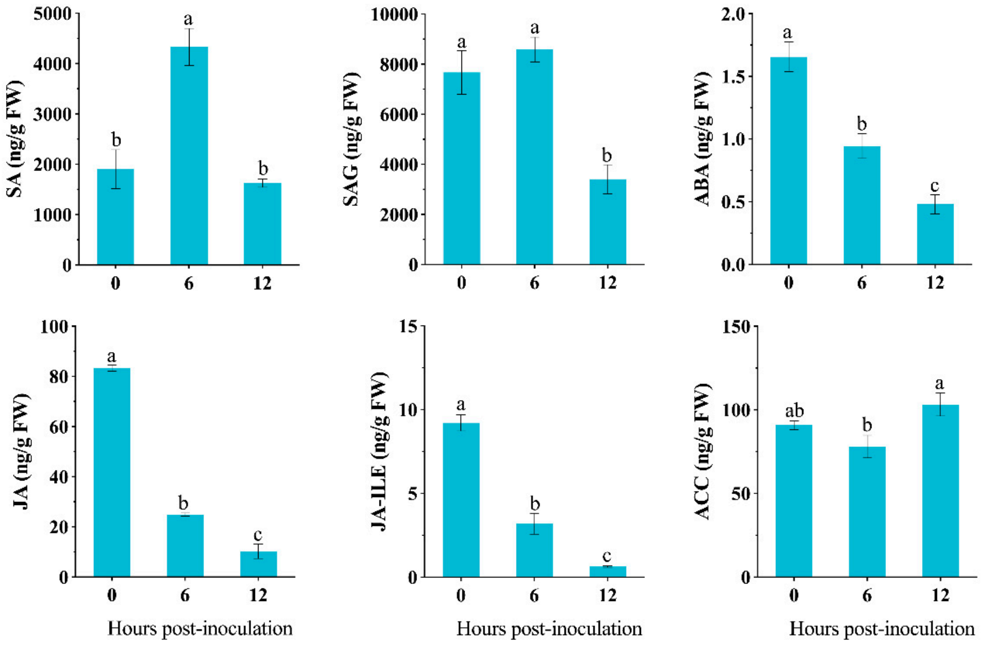

Figure S1.

The contents of salicylic acid (SA), SA-2-O-β-glucoside (SAG), jasmonic acid (JA), jasmonoyl-isoleucine (JA-ILE), abscisic acid (ABA), and 1-aminocyclopropane-1-carboxylic acid (ACC) were detected at five time points after inoculation with H. parasitica (0, 6, and 12 hpi). Different letters indicate significant differences based on post-hoc Tukey's test (Analysis of Variance $P < 0.05$).
